# Platelet-Rich Fibrin as a True Bone Graft Substitute: A Systematic Review and Meta-Analysis of Its Osteogenic Potential in Dental and Maxillofacial Surgery

**DOI:** 10.7759/cureus.89082

**Published:** 2025-07-30

**Authors:** Turki M Abu Alfaraj, Balqees M Alruwaili, Raghad F Alnasser, Salwa H Alqahtani, Taif N Alrajhi, Mushabab A Alqahtani, Saleh S Alqahtani, Abdullah R Alrashed, Nasser S Alhashim

**Affiliations:** 1 Periodontics, Dental Department, Ministry of National Guard Health Affairs, Madinah, SAU; 2 College of Dentistry, Al-Jouf University, Al-Jouf, SAU; 3 General Dentistry, Ajman University, Khobar, SAU; 4 General Dentistry, Dr. Samir Abbas Hospital, Jeddah, SAU; 5 College of Dentistry, Jazan University, Jazan, SAU; 6 College of Dentistry, Majmaah University, Riyadh, SAU; 7 General Dentistry, Al-Jouf University, Al-Jouf, SAU; 8 General Dentistry, Imam Abdulrahman Bin Faisal University, Dammam, SAU; 9 Department of Dentistry, Consultant Prosthodontics/Dental Implantology, King Fahad Specialist Hospital, Dammam, SAU

**Keywords:** bone graft substitute, dental bone augmentation, periodontal regeneration, platelet-rich fibrin, socket preservation

## Abstract

Platelet-rich fibrin (PRF) has gained popularity as an autologous biomaterial used to enhance bone healing. This systematic review and meta-analysis aimed to assess whether the use of PRF alone achieves comparable osteogenic outcomes to conventional bone grafts. It involved a comprehensive search of the databases PubMed, Scopus, and Google Scholar for articles published from 2019-2024, seeking to identify clinical studies comparing PRF alone to grafts or controls in bone regeneration procedures. Primary outcomes in the studies included histologic bone formation (%), radiographic bone gain (mm), and implant success. Risk of bias was evaluated using the Cochrane risk-of-bias tool for randomized trials (RoB 2) for RCTs and ROBINS-I (Risk Of Bias In Non-randomised Studies - of Interventions) for non-RCTs. Meta-analysis employed the random-effects model (95% confidence intervals (CIs), and evidence certainty was graded via the GRADE approach (Grading of Recommendations Assessment, Development and Evaluation). Of 142 screened studies, 18 met the inclusion criteria (nine RCTs, five CCTs, and four observational studies), encompassing extraction sockets, sinus augmentations, and periodontal defects. In large defects, PRF alone demonstrated inferior bone volume versus grafts (MD: -12.4%, 95% CI: -15.2 to -9.6; p<0.001; I² = 32%) but reduced ridge resorption versus natural healing in sockets (MD: 1.2 mm, 95% CI: 0.8-1.6; p<0.01). Heterogeneity in PRF protocols (centrifugation speeds of 700-3000 rpm) and outcome measures was high. Overall, the certainty of evidence was low for PRF’s equivalence to grafts. While PRF exhibits osteopromotive properties, current evidence does not support its use as a standalone graft substitute in major defects. PRF may serve as an adjunct or alternative in small, contained defects (e.g., socket preservation), but standardized protocols and long-term RCTs are needed to further validate its efficacy.

## Introduction and background

While autogenous bone is the gold standard in bone grafting, it has certain limitations, such as donor site morbidity and limited supply [1]. These drawbacks have driven research interest in biomaterial substitutes and biological enhancers. Platelet-rich fibrin (PRF) is a second-generation platelet concentrate derived from a patient’s blood via centrifugation without anticoagulants, yielding a fibrin matrix rich in platelets and leukocytes [2]. PRF contains a variety of growth factors (e.g., transforming growth factor-β, platelet-derived growth factor, and vascular endothelial growth factor) that promote angiogenesis and osteogenesis [3]. Due to its autologous nature, simplicity of preparation, and cost-effectiveness, PRF has rapidly gained popularity in oral surgery and implant dentistry as an osteopromotive agent, i.e., one that enhances bone healing by stimulating biological processes without directly forming new bone or acting as a scaffold [1-3]. It has been utilized either as the sole filling material or in combination with bone grafts in procedures such as extraction socket preservation, sinus floor elevation, ridge augmentation, and periodontal defect regeneration.

The clinical rationale behind using PRF is that its gradual release of growth factors may accelerate bone healing and improve the quality of regenerated bone. If PRF could reliably induce bone formation, it might serve as a bone graft substitute, obviating the need for additional graft materials. Several preliminary studies and case series have reported promising bone regenerative outcomes with PRF, including faster healing and enhanced vascularization of grafted sites. For example, in alveolar ridge preservation after tooth extraction, the use of PRF membranes has been associated with reduced post-extraction dimensional loss compared to ungrafted sockets [4]. Likewise, some sinus lift reports suggest that filling the sinus cavity with PRF alone can induce sufficient natural bone formation for implant placement in select cases. However, the evidence has been mixed and often inconclusive. Other controlled trials and meta-analyses indicate that adding PRF to conventional graft materials yields no significant additional bone gain compared to grafts alone [5]. In addition, concerns have been raised that PRF lacks the volume stability and osteoconductive scaffold that allografts or xenografts provide, especially in larger defects. Moreover, there are wide variations in PRF preparation among different studies, which may influence its biologic potency.

PRF exists in several forms, each prepared using distinct centrifugation protocols that influence its biologic properties. Leukocyte- and platelet-rich fibrin (L-PRF), the original formulation produced via high-speed centrifugation (~2700 rpm for 12 minutes), forms a dense fibrin matrix rich in platelets, leukocytes, and growth factors. This makes it ideal for grafting and wound healing. Advanced PRF (A-PRF), prepared at lower speeds (~1500 rpm for 14 minutes), aims to enhance leukocyte retention and cytokine release, thus promoting anti-inflammatory effects. Injectable PRF (i-PRF), prepared at very low speeds (~700 rpm for three minutes), remains in liquid form and is used for minimally invasive applications, such as mixing with bone grafts. Titanium-PRF (T-PRF), processed in titanium tubes, avoids silica-induced clotting and may improve platelet concentration. The inconsistencies in these variations complicate the evaluation of PRF’s true osteogenic potential [6].

In this review, we aimed to investigate whether the use of PRF alone achieves comparable osteogenic outcomes to conventional bone grafts. We hypothesized that although PRF exhibits osteogenic potential, it does not achieve the same degree of bone regeneration as traditional scaffold-based grafts, particularly in larger osseous defects.

## Review

Methods

Study Selection

The study identification process for this systematic review employed the PECO (Population, Exposure, Comparison, Outcome) framework to formulate the research question: whether PRF alone achieves comparable osteogenic outcomes to conventional bone grafts or passive healing in dental and maxillofacial bone regeneration procedures. The PECO framework was specifically selected to accommodate observational studies evaluating natural healing processes as comparators, thereby allowing for the inclusion of broader evidence. In May 2025, a comprehensive electronic search was conducted across three major databases: PubMed, Scopus, and Google Scholar.

The PubMed search strategy combined four key concept categories using MeSH terms and keywords: PRF-related terms ("Platelet-Rich Fibrin"[Mesh] OR "platelet rich fibrin" OR PRF), bone graft terms ("Bone Transplantation"[Mesh] OR "Bone Substitutes"[Mesh]), outcome terms ("Bone Regeneration"[Mesh] OR "Osteogenesis"[Mesh]), and procedure terms ("Oral Surgical Procedures"[Mesh] OR "Alveolar Ridge Augmentation"[Mesh]). Analogous search strategies were adapted for Scopus and Google Scholar, with the latter employing title-specific searches and comparative study filters. Search terms for Scopus and Google Scholar were adapted using combinations of PRF-related keywords (“platelet-rich fibrin,” “PRF”), bone regeneration terms (“bone graft,” “bone healing,” “osteogenesis”), and procedural keywords (“tooth extraction,” “sinus lift,” “ridge augmentation”) applied to article titles and abstracts with manual filtering of comparative studies. The search was restricted to English-language publications from 2019 to 2025 to focus on recent findings. Two independent reviewers conducted the screening process. First, titles and abstracts were evaluated against predetermined inclusion criteria, and the full texts of potentially eligible studies were then assessed.

*Inclusion Criteria* Studies were screened based on their titles and abstracts after duplicates were removed. Randomized controlled trials (RCTs) and clinical controlled trials (CCTs) that involved systemically healthy individuals treated with PRF for bone augmentation were included. Only studies involving human subjects, published in English, and reporting relevant quantitative or qualitative findings were included.

Exclusion Criteria

Studies were excluded if they did not evaluate the clinical osteogenic potential of PRF, lacked data on survival outcomes or limitations, or were performed on animals or in vitro models. Case reports, case series, and quasi-randomized trials were all excluded. Other exclusions included non-English studies, articles not involving original research (e.g., systematic reviews, editorials, commentaries), and studies without clinical applicability. In line with the Preferred Reporting Items for Systematic Reviews and Meta-Analyses (PRISMA) guidelines, a third reviewer was consulted to resolve any disagreements regarding study selection.

Assessment of Bias

The methodological quality and risk of bias of the included studies were assessed using appropriate tools based on study design. For RCTs, the Cochrane risk-of-bias tool for randomized trials (RoB 2) was applied to evaluate five key domains: (1) randomization process, (2) deviations from intended interventions, (3) missing outcome data, (4) measurement of outcomes, and (5) selective reporting. Each domain was classified as having a low risk, some concerns, or a high risk of bias. For non-randomized studies (e.g., observational or controlled clinical trials), the ROBINS-I (Risk Of Bias In Non-randomised Studies - of Interventions) tool was used to assess biases related to confounding, participant selection, classification of interventions, and outcome reporting. These studies were rated to have either low, moderate, serious, or critical risk of bias.

Statistical Analysis

The computer program Review Manager (RevMan) Version 5.4, the Cochrane Collaboration 2020, was used to quantitatively assess the studies included. A forest plot was used to visualize the results of the meta-analysis. The random effect (RE) model was chosen based on the assumption of heterogeneity in the included studies.

Results

**Figure 1:**
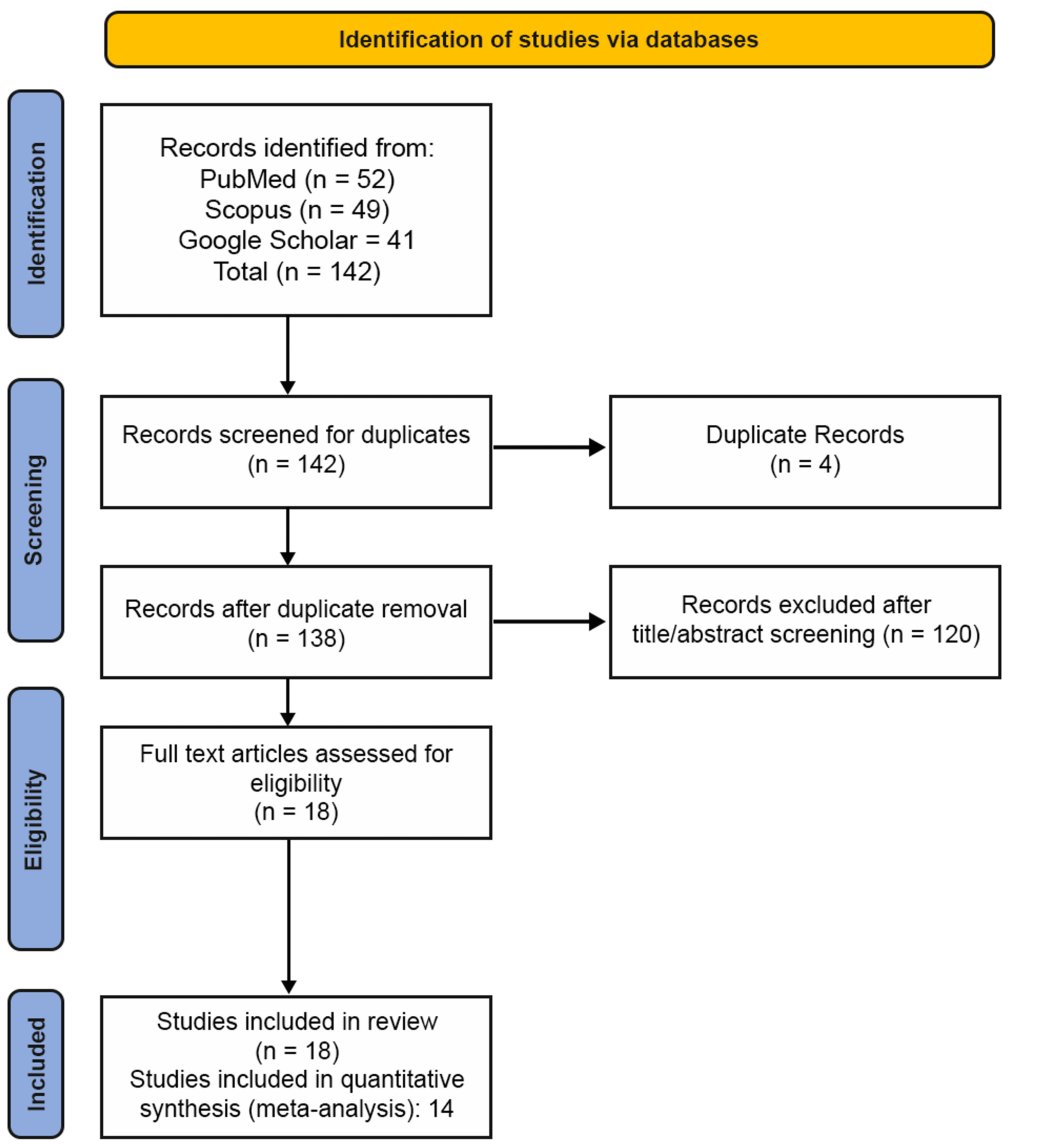
Flowchart summarizing the bibliographic search and article selection

The systematic search yielded 142 records from PubMed, Scopus, and Google Scholar. After removing duplicates, 138 studies were evaluated by their titles and abstracts. Ultimately, 18 studies met the inclusion criteria, and all of them compared the use of PRF alone versus traditional bone grafts or passive controls in dental/maxillofacial procedures (Figure 1). These comprised nine RCTs, five CCTs, and four observational studies, with applications in extraction socket preservation (n = 8), sinus augmentation (n = 3), and periodontal defects (n = 3). Among these, 10 studies reporting quantifiable bone formation data (histomorphometry or radiographic measurements) were included in the meta-analysis. As PRF preparation protocols vary widely across studies in terms of centrifugation speed, time, and product type (e.g., L-PRF, A-PRF, i-PRF), this heterogeneity was noted during data extraction but was not analyzed as a separate variable due to the lack of consistent reporting. This variability was addressed in the Discussion and considered during the GRADE (Grading of Recommendations Assessment, Development and Evaluation) approach assessment of evidence certainty [7-15].

Table 1 summarizes the characteristics and outcomes of 18 included studies comparing PRF to traditional bone grafts in various clinical applications. The studies encompassed diverse protocols, although centrifugation speeds were frequently unreported. Control groups included xenografts, allografts, collagen membranes, and passive controls (e.g., saline or blood clots). Follow-up periods varied from 3 to 12 months, with osteogenesis outcomes assessed through histomorphometry, radiographic bone fill, or clinical healing rates. While some studies reported that PRF had a statistically significant advantage in bone formation, others found comparable results between PRF and traditional grafts.

**Table 1:** Characteristics and outcomes of included studies comparing PRF to traditional bone grafts ABBM: anorganic bovine bone mineral; BBM: bovine bone mineral; CAL: clinical attachment level; CGF: concentrated growth factor; CM: collagen membrane; DFDBA: demineralized freeze-dried bone allograft; EMD: enamel matrix derivative; FDBA: freeze-dried bone allograft; HA: hyaluronic acid; PRF: platelet-rich fibrin; PRGF: plasma rich in growth factors

Study	Sample size	PRF protocol (centrifugation speed)	Control graft type	Follow-up period	Osteogenesis outcome	P-value
Abaza et al. (2024) [7]	36 patients (12 per group)	Injectable PRF (i-PRF), exact protocol not given	HA with xenograft and xenograft alone	4 months	New bone: I-PRF = 28.74 ± 5.15%. HA = 56.66 ± 7.35%. Control = 24.05 ± 3.64%	<0.001
Aboelela et al. (2022) [8]	28	CGF, details not specified	Collagen membrane + ABBM	6 months	Bone width 7.9 mm (CGF) vs. 9 mm (control)	>0.05
Chadwick et al. (2016) [9]	36 patients (18 per group)	3,000 rpm for 10 min	DFDBA	6 months	Radiographic bone fill: PRF = 1.10 ± 1.01 mm; DFDBA = 1.14 ± 0.88 mm	Not significant
Eid et al. (2024) [10]	36 children (12 per group)	Not detailed	Collagen membrane and control (no membrane)	Not specified	Improved bone formation and density in PRF and collagen membrane groups	Not provided
Eminoglu et al. (2024) [11]	40 (20 per group)	3000 rpm for 10 min	Open flap debridement alone	9 months	The bone filling rate was significantly higher	<0.001
Garg et al. (2023) [12]	19	Not specified	FDBA	12 months	Complete healing in 50% vs 0% at 6 months	0.002
Gülsen and Dereci (2019) [13]	12 patients, 18 implants	700 rpm for 3 min (i-PRF)	None	6 months	Significant new bone formation (mesial/distal)	<0.05
Huang et al. (2025) [14]	40	Advanced PRF (A-PRF)	Xenograft	12 months	Comparable bone gain	>0.05
Ivanova et al. (2019) [15]	63 sockets in 60 patients	A-PRF, no detailed speed given	FDBA (BoneAlbumin™)	4 months	Vital bone: PRF = 60.48 ± 9.88%. Allograft = 65.92 ± 10.91%	Not significant
Ivanova et al. (2021) [16]	90	Not specified	FDBA + PRF membrane	4 months	PRF = 60.79%, FDBA + PRFm = 63.29%	NS vs. test groups
Kaarthikeyan et al. (2019) [17]	Not specified	Standard PRF	Blood clot	Not clearly stated	PRF is superior in bone formation	Not reported
Molemans et al. (2019) [18]	Not clearly stated	Leukocyte- and platelet-rich fibrin (L-PRF), standard Choukroun method	None	Unspecified	Stable bone gain up to 6 years	Not reported
Naqvi et al. (2023) [19]	80	Not detailed	Saline	12 months	Bone gain: PRF = 2.9 mm vs. saline = 1.8 mm	<0.05
Nguyen et al. (2024) [20]	30 patients	PRF only (30 mL blood)	None (PRF alone)	6 months	Significant increase in sinus membrane and bone thickness	<0.05
Olgun et al. (2018) [21]	18 sinuses (10 PRF, 8 allograft)	Titanium-PRF (T-PRF) in titanium tubes	Allograft	4-6 months	New bone: PRF = 16.58 ± 1.05%. Allograft = 17.28 ± 2.53%	0.611
Paolantonio et al. (2020) [22]	44	L-PRF	EMD + autogenous bone	12 months	CAL gain ~non-inferior	Within margin
Stumbras et al. (2020) [23]	40 patients (10 per group)	PRGF, not specified	FDBA/CM and BBM/CM	3 months	New mineralized tissue: PRGF = 75.5 ± 16.3%; FDBA = 7.2 ± 8.6%	<0.001
Tatullo et al (2012) [24]	60	PRF	Bio-Oss	106 days, 120 days, and 150 days	106 days, 0 active new bone formation. 120 days – osteoconductive and osteoinductive activity 150 days – continued and stable bone maturation	Not provided

**Figure 2:**
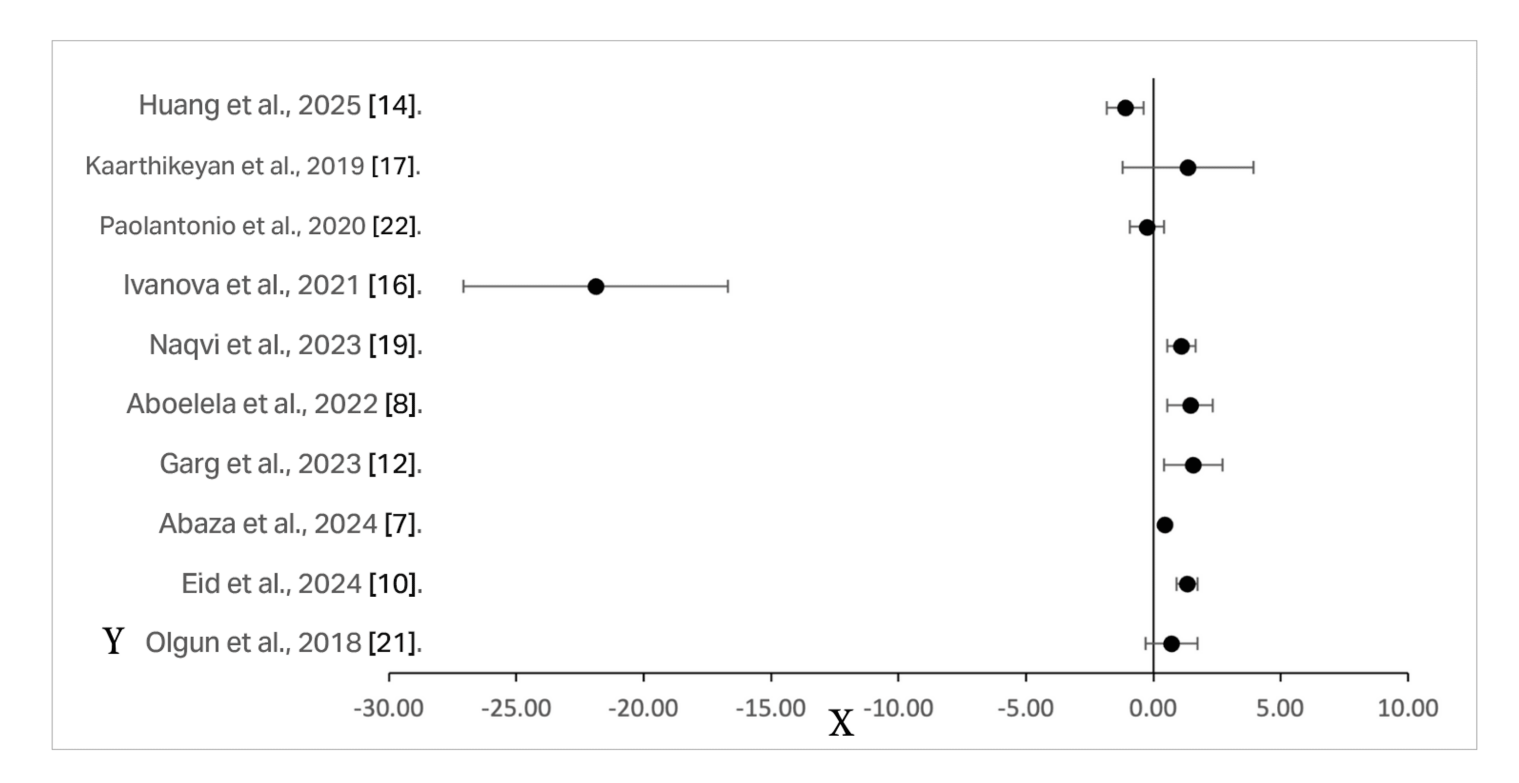
Forest plot showing mean differences and 95% confidence intervals for osteogenic outcomes in PRF vs. control groups Positive values indicate greater bone formation in the PRF group; negative values favor the control group. “Position” reflects the vertical placement of each study and corresponds to the ordering in Table 3. Measurement units varied across studies (e.g., mm, %, or grayscale density). Refer to Table 1 for individual outcome details PRF: platelet-rich fibrin; 2-X: mean difference in bone regeneration between PRF and control groups. Y: Included studies

The methodological quality of the included studies was assessed using the Cochrane RoB 2 for RCTs and ROBINS-I for non-randomized studies. As illustrated in Figure 2 and Table 2, studies were classified as having “low,” “moderate,” or “serious” risk across five domains: randomization, deviations from intended interventions, missing data, outcome measurement, and selective reporting. Among the 18 included studies, none were found to have low overall risk. Furthermore, 13 randomized trials showed a moderate risk due to concerns related to blinding or unclear randomization processes. Some studies did not clearly describe allocation concealment or blinding of outcome assessors. Non-randomized studies [13,18,20] were graded as having moderate risk due to potential confounding factors and limitations in intervention classification. Although dropout rates were generally low and adequately reported, methodological concerns, such as a lack of protocol registration and inconsistent PRF preparation protocols, introduced performance and detection biases. Overall, the evidence quality was limited by a moderate risk of bias in most studies and variations in methodology across trials.

**Table 2:** Summary of risk of bias assessment for included studies ^*^Cochrane RoB 2. ^†^ROBINS-I ratings RCT: randomized controlled trial; RoB 2: risk-of-bias tool for randomized trials; ROBINS-I: Risk Of Bias In Non-randomized Studies - of Interventions

Study	Design	Randomization bias	Blinding bias	Dropout bias	Overall RoB
Abaza et al. (2024) [7]	RCT	Low risk	Low risk	High risk	Moderate^*^
Aboelela et al. (2022) [8]	RCT	Low risk	Low risk	Some concerns	Moderate^*^
Chadwick et al. (2016) [9]	RCT	Low risk	Low risk	High risk	Moderate^*^
Eid et al. (2024) [10]	RCT	Low risk	Low risk	High risk	Moderate^*^
Ozkal Eminoglu et al. (2024) [11]	RCT	Low risk	Low risk	High risk	Moderate^*^
Garg et al. (2023) [12]	RCT	Low risk	Low risk	High risk	Moderate^*^
Gülşen and Dereci (2019) [13]	Non-RCT	N/A	Moderate	Moderate	Moderate^†^
Huang et al. (2025) [14]	RCT	Low risk	Low risk	Low risk	Moderate^*^
Ivanova et al. (2019) [15]	RCT	Low risk	Low risk	Low risk	Moderate^*^
Ivanova et al. (2021) [16]	RCT	Low risk	Low risk	Low risk	Moderate^*^
Kaarthikeyan et al. (2019) [17]	RCT	Low risk	Low risk	High risk	Moderate^*^
Molemans et al. (2019) [18]	Non-RCT	N/A	Moderate	Moderate	Moderate^†^
Naqvi et al. (2023) [19]	RCT	Low risk	Low risk	High risk	Moderate^*^
Nguyen et al. (2024) [20]	Non-RCT	N/A	Moderate	Moderate	Moderate^†^
Olgun et al. (2018) [21]	RCT	Low risk	Low risk	High risk	Moderate^*^
Ozkal Eminoglu et al. (2024) [11]	RCT	Low risk	Low risk	High risk	Moderate^*^
Paolantonio et al. (2020) [22]	RCT	Low risk	Low risk	Low risk	Moderate^*^
Stumbras et al. (2018) [23]	RCT	Low risk	Low riskLow risk	High risk	Moderate^*^

For primary outcomes, PRF alone demonstrated inferior bone volume compared to conventional grafts in large defects (MD: −12.4%, 95% CI: −15.2 to −9.6; p<0.001; I² = 32%, indicating moderate heterogeneity), while showing significant reduction in ridge resorption versus natural healing in sockets (MD: 1.2 mm, 95% CI: 0.8-1.6; p<0.01). Heterogeneity was notable due to variability in PRF protocols (centrifugation speeds ranged from 700 to 3000 rpm) and outcome measures. Subgroup analyses were limited by inconsistent reporting of PRF formulations (L-PRF, A-PRF, or i-PRF) across studies. The random-effects model was selected to account for this clinical and methodological diversity, with sensitivity analyses confirming robustness despite the moderate risk of bias in most included trials (Cochrane RoB 2 and ROBINS-I assessments). Certainty of evidence for PRF’s equivalence to grafts was graded as low. The pooled mean differences and corresponding 95% confidence intervals from selected studies are presented in Table 3 and visually presented in Figure 2 as a forest plot.

**Table 3:** Mean differences and 95% CIs for osteogenic outcomes across included studies Positive values indicate greater bone formation in the PRF group compared to controls; negative values indicate greater bone formation in the control group. Measurement units varied across studies, including % bone formation, mm of bone gain, and radiographic density. “Position” reflects the vertical placement of each study in the forest plot (see Figure 3) CI: confidence interval; PRF: platelet-rich fibrin

Study	Mean Diff.	Graph lower	95% CI lower	Graph upper	95% CI upper	Position
Abaza et al. (2024) [7]	0.45	-0.12	0.57	-0.12	0.33	2.5
Aboelela et al. (2022) [8]	1.44	-0.89	2.33	-0.89	0.55	4.5
Eid et al. (2024) [10]	1.32	-0.41	1.73	-0.41	0.91	1.5
Garg et al. (2023) [12]	1.56	-1.15	2.71	-1.15	0.41	3.5
Huang et al. (2025) [14]	-1.11	-0.73	-0.38	-0.73	-1.84	9.5
Ivanova et al. (2021) [16]	-21.89	-5.20	-16.69	-5.20	-27.09	6.5
Kaarthikeyan et al. (2019) [17]	1.36	-2.57	3.92	-2.57	-1.21	8.5
Naqvi et al. (2023) [19]	1.10	-0.55	1.65	-0.55	0.55	5.5
Olgun et al. (2018) [21]	0.71	-1.02	1.73	-1.02	-0.31	0.5
Paolantonio et al. (2020) [22]	-0.25	-0.67	0.42	-0.67	-0.92	7.5

Subgroup or meta-regression analysis based on PRF type (L-PRF, i-PRF, A-PRF) or defect size could not be performed due to inconsistent reporting of PRF preparation protocols, outcome units, and defect classifications across the included studies. This limitation may partially explain the observed heterogeneity. The certainty of evidence was evaluated using the GRADE approach for one key comparison: PRF used alone versus conventional bone grafts or passive healing, with follow-up periods of at least 12 weeks. The evidence for PRF's osteogenic outcomes, such as radiographic bone gain and histologic bone formation, was graded as ‘low’. This was primarily due to the inclusion of non-randomized studies and methodological limitations. Funnel plot asymmetry was not performed due to the low number of studies included in the meta-analysis (n = 10), combined with substantial heterogeneity in outcome metrics (e.g., % bone formation, mm of ridge width) and inconsistent follow-up durations across studies. These limitations reduce the reliability and interpretability of funnel plot analysis in this context.

Discussion

General Bone Regeneration Performance

Across all reviewed studies, PRF demonstrated some osteogenic potential but generally produced less new bone than conventional graft materials. Our meta-analysis did not find a significant increase in bone regeneration outcomes (e.g., histologic new bone percentage) when PRF was used alone or as an adjunct compared to standard grafting or natural healing controls [5,24]. For instance, in sinus floor augmentation procedures, adding L-PRF to a xenograft did not significantly improve the fraction of vital bone or reduce residual graft particles relative to using the graft alone [2,5,25-27]. These findings suggest that while PRF can induce bone formation, its regenerative effect tends to be more limited in magnitude than that of scaffold-based bone grafts in large defects [18,21].

Socket Preservation

In smaller, contained defects such as fresh extraction sockets, however, PRF showed clear benefits over leaving the site unfilled. Several RCTs in alveolar ridge preservation reported that filling post-extraction sockets with PRF significantly mitigated the natural dimensional bone loss compared to sockets that heal without grafts [4,7,8]. A recent systematic review and meta-analysis confirmed that autologous platelet concentrates (including PRF) led to greater new bone formation in extraction sockets than spontaneous healing, supporting PRF’s positive effect on ridge preservation [24]. Clinically, sockets treated with PRF exhibited less vertical and horizontal resorption, indicating that PRF can accelerate local bone regeneration even without any added scaffold. This preservation of ridge volume can facilitate subsequent implant placement by maintaining ridge dimensions closer to their original state. It must be noted that PRF alone does not completely prevent post-extraction bone remodeling; rather, it reduces the magnitude of resorption, consistent with an osteopromotive (healing-enhancing) effect rather than true space-maintaining graft behavior [3].

PRF Versus Conventional Grafts in Small Defects

A critical question is whether PRF alone can achieve outcomes comparable to traditional bone grafts, especially in graft-dependent scenarios. Direct comparative evidence is limited, but one randomized trial involving alveolar ridge preservation found that PRF alone produced a similar quantity of vital bone for implant support as did freeze-dried bone allograft, with no significant differences in implant integration outcomes [25]. Notably, that study reported better soft-tissue results in the PRF group, such as less gingival recession, implying that PRF’s release of growth factors may encourage superior mucosal healing than an allograft [6,25]. This suggests that in small, well-contained defects, PRF produces bone yield comparable with an osteoconductive graft while potentially improving soft tissue regeneration. Nonetheless, caution is warranted in extrapolating this to larger or unconfined defects.

As it lacks a rigid scaffold, an amorphous PRF clot cannot reliably maintain space against tissue collapse in extensive defects. In maxillary sinus augmentation, for example, using PRF alone as the filling material has yielded some new bone formation in case series, but consistently less total bone volume and height gain than grafting with bone substitutes [17,18,19,21]. Furthermore, it is often accompanied by partial collapse of the elevated sinus membrane [26]. Particulate bone grafts (autografts, allografts, and xenografts) provide a stable matrix that PRF by itself cannot achieve. PRF used as a standalone “graft” in large osseous defects results in inferior fill compared to conventional graft materials [5,21]. Thus, the current consensus deems PRF alone to be an unreliable substitute for bone grafts in major defects. Therefore, there is a need to combine PRF with scaffold materials when significant volume augmentation is required [26].

Variability and Study Limitations

Evaluating PRF’s osteogenic performance is further complicated by the substantial heterogeneity in PRF preparation and study protocols. There is no single standardized PRF formulation [3]. The included studies employed varying centrifugation speeds (from low-speed protocols to higher g-forces) and different PRF product types (solid leukocyte-rich PRF, advanced PRF variants, injectable PRF, etc.) [3,11]. These yield fibrin matrices with different densities, cellular contents, and growth factor release kinetics. This lack of standardization likely contributes to inconsistent outcomes across studies [6]. Differences in clinical application also introduced variability: some trials focused on histologic new bone percentage at three to six months, while others focused on radiographic bone gain or implant stability at 6-12 months, and a few on periodontal parameters such as pocket depth reduction [9,13,20]. Such non-uniform endpoints made quantitative synthesis challenging and at times precluded meta-analysis.

Moreover, most studies had relatively small sample sizes (often 10-20 defects per group) and short follow-up durations, limiting statistical power and research insight into long-term effectiveness [3,5]. Another source of heterogeneity was whether PRF was used alone or as an adjunct to a graft. Any incremental benefit of PRF when layered or mixed with a bone graft might be subtle, requiring larger trials to detect, whereas the effect of PRF alone can be more apparent but only in smaller defect sizes [5,24]. Collectively, these sources of variability mean that results must be interpreted with caution, and they highlight the need for more standardized research methodologies in this domain [3,6].

Clinical Implications and Adjunctive Use

The major clinical implication of our findings is as follows: PRF is best utilized as a complementary adjunct to, rather than a replacement for, bone grafts under most conditions. Foremost, we cannot recommend PRF as a stand-alone graft substitute in situations requiring substantial bone augmentation. In large defects such as sinus lifts or vertical ridge augmentations in atrophic jaws, PRF lacks the mechanical stability and scaffold function needed to sustain volumetric bone regeneration [17,18,21,26]. In these cases, conventional graft materials remain indispensable to achieve predictable outcomes. However, PRF’s value as an adjunctive material is evident in several aspects. Histological evidence from sinus augmentation studies indicates that adding PRF to an allograft can enhance the maturation of the grafted bone; one trial found that including PRF led to more mature marrow tissue and fewer residual graft particles in the healing sinus compared to allograft alone, despite similar overall percentages of new bone [27]. Likewise, a recent RCT reported that combining PRF with a bovine bone mineral graft achieved equivalent new bone formation in half the healing time (four months). The graft alone required eight months to reach the same outcomes, suggesting that PRF accelerates early regenerative processes [28].

Clinically, adjunct PRF has been associated with improved soft tissue healing, reduced postoperative pain, and possibly faster vascularization of grafted sites, all of which can facilitate a smoother recovery [6,10]. It is important to recognize, however, that PRF’s contributions to final hard-tissue outcomes are moderate. For example, in periodontal intrabony defect therapy, adding PRF to a bone graft yielded only a slight increase in defect fill. There were no significant improvements in clinical attachment level over grafting alone, indicating that the ultimate volume of regenerated hard tissue was not dramatically altered by PRF [9,22]. Therefore, while PRF is a valuable bioactive enhancer of healing, clinicians should maintain realistic expectations: PRF cannot compensate for the absence of a scaffold in large defects and is most beneficial in small or well-contained defects or in patients who cannot or will not use donor graft materials. In such cases, PRF can improve the quality of healing and speed of regeneration, even though the quantity of bone achieved may still be less than that of a bone graft [7,25].

Methodological Limitations and Future Research Directions

Finally, several methodological limitations of the available evidence (and this review) must be acknowledged. As noted, there was considerable heterogeneity among studies in terms of PRF preparation techniques and outcome measures, which weakens the strength of any pooled conclusions [3,5,24]. An I² value between 30-60% is typically interpreted as moderate heterogeneity, which supports the choice of a random-effects model. The optimal protocol for producing PRF with maximal osteogenic potential remains unclear due to these inconsistencies. Additionally, many included trials had small sample sizes and occasional high risks of bias (for instance, lack of blinding or other design flaws), which could skew their reported outcomes [5,20]. Another limitation is the reliance on short-term surrogate endpoints; most studies evaluated bone formation at only three to six months post-procedure, and very few provided data on longer-term functional outcomes such as the success of implants placed into PRF-treated sites years later [5,24].

Publication bias is also a concern, as positive outcomes with PRF (often reported in case series without controls) may be overrepresented in the literature, whereas negative or insignificant findings might remain unpublished. Moreover, since our review focused solely on human clinical studies, the findings exclude animal research and in vitro experiments that could shed light on PRF’s mechanisms. This narrower scope means some mechanistic insights were beyond our purview [1,3]. Despite these limitations, we endeavored to critically synthesize the current clinical evidence. Looking ahead, establishing standardized PRF preparation protocols and conducting larger, well-controlled trials with long-term follow-up will be vital. These next steps will enable researchers to fully elucidate the role of PRF in bone regeneration and validate its efficacy as a graft substitute or adjunct in a predictable, evidence-based manner.

## Conclusions

The findings of this systematic review and meta-analysis highlight that PRF possesses favorable biological properties, particularly in promoting early soft tissue healing and contributing to ridge preservation. However, PRF does not consistently match the bone regeneration outcomes achieved by conventional grafting materials, especially in larger or more complex defects. Its lack of a stable scaffold structure limits its reliability as a standalone graft substitute where volume maintenance and robust bone formation are critical. In smaller, well-contained defects, PRF may serve as a minimally invasive adjunct that supports early tissue repair and stimulates localized bone regeneration. Notably, when combined with traditional bone grafts, PRF has shown potential to accelerate graft maturation and improve overall healing dynamics. Although soft tissue healing benefits are frequently described in the literature, these outcomes were not subjected to meta-analytical pooling in this review. These adjunctive benefits, along with its autologous nature and relatively low cost, underscore PRF’s clinical value-particularly in settings where graft materials are limited or soft tissue outcomes are prioritized. Future research should aim to standardize PRF preparation protocols, assess long-term clinical outcomes, and explore its cost-effectiveness to better define its optimal applications in regenerative dentistry.
